# Comprior: facilitating the implementation and automated benchmarking of prior knowledge-based feature selection approaches on gene expression data sets

**DOI:** 10.1186/s12859-021-04308-z

**Published:** 2021-08-12

**Authors:** Cindy Perscheid

**Affiliations:** Hasso Plattner Institute, Digital Engineering Faculty, University of Potsdam, Potsdam, Germany

**Keywords:** Feature selection, Prior knowledge, Gene expression, Reproducible benchmarking

## Abstract

**Background:**

Reproducible benchmarking is important for assessing the effectiveness of novel feature selection approaches applied on gene expression data, especially for prior knowledge approaches that incorporate biological information from online knowledge bases. However, no full-fledged benchmarking system exists that is extensible, provides built-in feature selection approaches, and a comprehensive result assessment encompassing classification performance, robustness, and biological relevance. Moreover, the particular needs of prior knowledge feature selection approaches, i.e. uniform access to knowledge bases, are not addressed. As a consequence, prior knowledge approaches are not evaluated amongst each other, leaving open questions regarding their effectiveness.

**Results:**

We present the Comprior benchmark tool, which facilitates the rapid development and effortless benchmarking of feature selection approaches, with a special focus on prior knowledge approaches. Comprior is extensible by custom approaches, offers built-in standard feature selection approaches, enables uniform access to multiple knowledge bases, and provides a customizable evaluation infrastructure to compare multiple feature selection approaches regarding their classification performance, robustness, runtime, and biological relevance.

**Conclusion:**

Comprior allows reproducible benchmarking especially of prior knowledge approaches, which facilitates their applicability and for the first time enables a comprehensive assessment of their effectiveness.

**Supplementary Information:**

The online version contains supplementary material available at 10.1186/s12859-021-04308-z.

## Background

Benchmarking is essential to show the effectiveness of analysis methods in a broader context and allows to draw conclusions regarding their practicability, usefulness, reliability, and robustness. In the context of feature selection on gene expression data sets, there is only limited support for automated benchmarking. Suitable tools are either not extensible, which is required for testing custom approaches, or do not support cross-validation strategies, which is crucial to prove approach robustness and stability [1–3]. Additionally, no tool addresses the specific needs for integrative analyses that incorporate prior biological knowledge into the analysis. So-called *prior knowledge approaches* integrate biological knowledge, e.g. on genes and their interactions, from public knowledge bases, e.g. Gene Ontology [4, 5]. It is assumed that prior knowledge approaches identify more robust and biologically meaningful biomarkers. However, current findings on their effectiveness only show relative improvements in a limited scope [6–10]: comparisons among prior knowledge approaches are sparse, cross-validation across data sets are rare, the choice and impact of the applied knowledge base is never discussed. The major obstacle for benchmarking prior knowledge approaches is the high implementation effort: heterogeneous data from knowledge bases must be mapped to a uniform format; cross-validation strategies, especially across data sets, must be set up. An infrastructure that allows to effortlessly implement and comprehensively evaluate custom feature selection approaches would enable researchers to efficiently develop and optimize novel prior knowledge approaches.

With this work, we present *Comprior*, our contribution to enable comprehensive and reproducible benchmarking of feature selection approaches, with a special focus on – but not limited to – prior knowledge approaches. Comprior provides an implementation and evaluation infrastructure that unifies knowledge base access and allows to comprehensively assess both prior knowledge and traditional feature selection approaches regarding their quantitative performance and biological relevance. Instead of being constrained by heterogeneous knowledge base information, data harmonization, and complex benchmark setups, researchers can now concentrate on the development of their own feature selection approach and flexibly combine it with multiple knowledge bases or statistical approaches. This work describes the technical details of Comprior including its architecture design, specification of selected functionality, and an example case study.

## Functionality

**Fig. 1:**
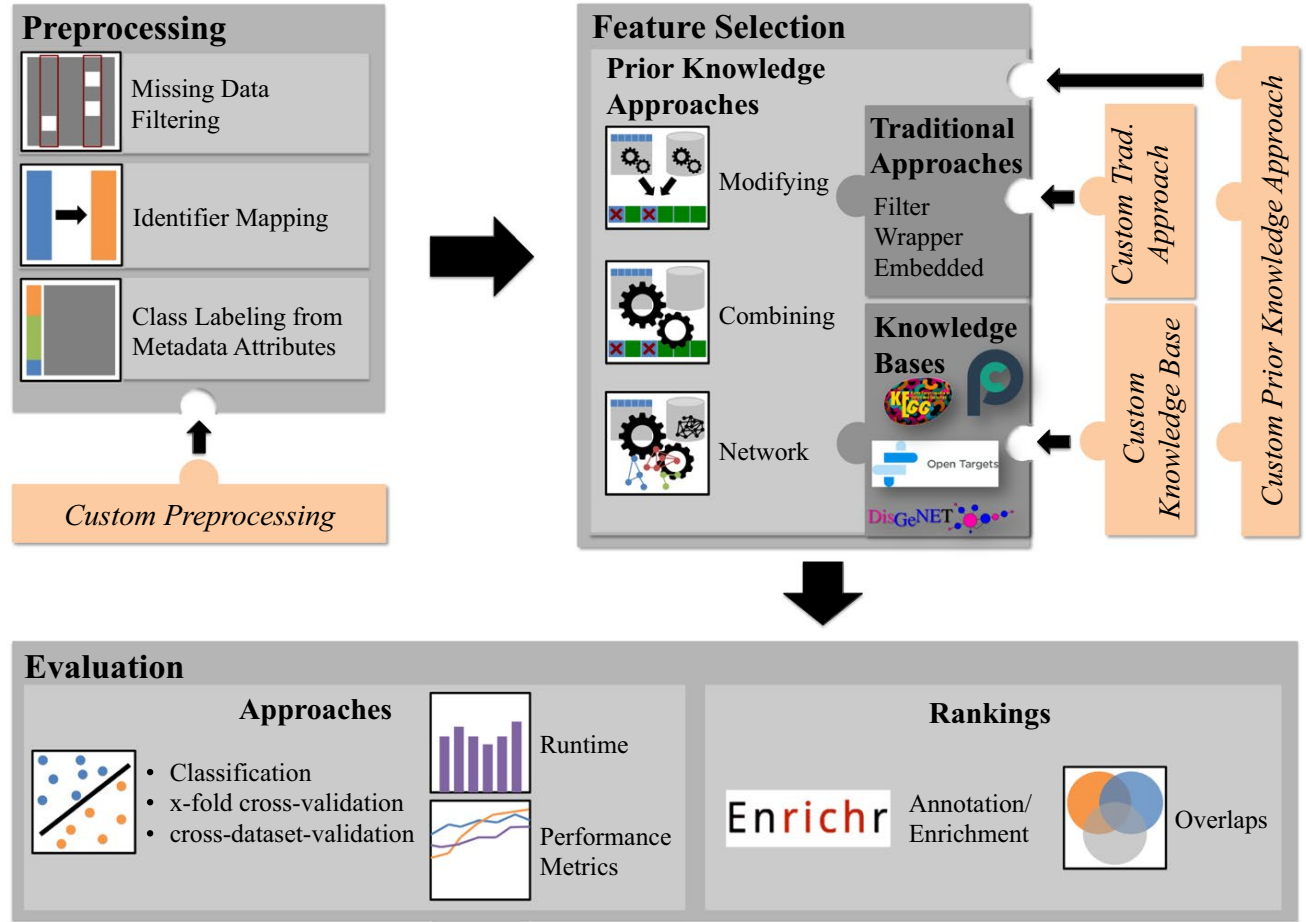
Comprior covers preprocessing, feature selection, and evaluation functionality. The modular design allows to easily extend Comprior by custom functionality. (Original figure created by Cindy Perscheid)

Comprior supports a broad range of the classical analysis workflow for feature selection and classification tasks, covering preprocessing, feature selection, and evaluation. Figure 1 provides an overview of Comprior’s functionality and its modular design. In the following, we go into detail on the most important functionalities.

For preprocessing, Comprior provides identifier mapping, data cleansing, and data labeling based on user-defined metadata attributes. Identifier mapping is carried out automatically throughout the whole analysis process using g:Profiler’s mapping service [11]. Input data can thus contain identifiers of genes, microarray probes, or similar, which can be mapped to any desired output format supported by g:Profiler. Input data can be filtered by samples and features that have missing values above a specified threshold. Input data is automatically labeled with a user-defined metadata attribute. Comprior can be extended by custom preprocessing functionality, e.g. normalization. From the given input data set, Comprior creates density plots, distribution box plots, and multi-dimensional scaling (MDS) plots for quality assessment. To assess knowledge base coverage, Comprior computes summary statistics for the available prior knowledge.

For feature selection, Comprior provides a broad range of both statistical and prior knowledge approaches^1^. Available statistical approaches cover filter, wrapper, and embedded approaches. Available prior knowledge approaches cover modifying, combining, and network approaches [12]. *Modifying* prior knowledge approaches are filtering or extension steps added before or after statistical feature selection. *Combining* prior knowledge approaches integrate prior knowledge more thoroughly into the feature selection process. Comprior currently provides two combining approaches. The first combining approach computes a feature relevance score by weighting a statistical relevance score $s_i^{trad}$, e.g. computed via variance or any other available traditional selection method, by an association score $s_i^{kb}$ retrieved from a knowledge base: $s_i = s_i^{trad} \times s_i^{kb}$ The second combining approach introduces prior knowledge as feature-specific penalty score during Lasso computation [13]. *Network* approaches incorporate networks, e.g. containing gene-gene interaction information, and map the input feature space, e.g. genes, to relevant networks. Comprior currently provides a network approach that selects relevant pathways from a knowledge base based on the strategy described by Tian et al. [14]: A pathway is considered relevant if the gene expression profiles of its member genes correlate with the data set classes. Corresponding feature values for the selected pathways can be computed either based on a pathway activity score as defined by Lee et al. or based on Vert and Kanehisa’s definition of pathway relevance and smoothness [9, 15]. All of these approaches can be flexibly combined with any of the currently available knowledge bases: KEGG, OpenTargets, DisGeNET, and PathwayCommons [16–19]^2^.

For evaluation, Comprior provides several options to assess the effectiveness, robustness, and biological relevance of feature sets. Users can select multiple standard classifiers for k-fold cross-validation to assess the effectiveness of a feature set. Classification results are assessed with standard measures, e.g. accuracy or $\hbox {F}_{1}$﻿. In addition, Comprior can carry out cross-validation of the selected features on a second data set for robustness evaluation. This second data set can be related to the original input data set in a traditional train-test manner, but also be completely unrelated. Runtime performance for the distinct feature selection approaches is measured as well. To assess the biological relevance of feature sets, Comprior uses Enrichr for gene set annotation and enrichment [20, 21]. Feature sets are compared to each other via overlaps (features, annotations, and enrichment) and Kendall’s W [22].

Wilkinson et al. proposed the Findable, Accessible, Interoperable, and Reusable (FAIR) principles for the management of digital assets [23]. While these principles were originally intended for the management of data sets, recent efforts are aiming at transferring and adapting them to software as well. Based on guidelines summarized by Gruenpeter et al., we discuss the software FAIRness of Comprior [24]. The complete software is licensed under the MIT licencse and freely accessible in a public GitHub repository that also provides a limited version control (F, A, R). Comprior can be installed from source in a semi-automated process or directly be executed in a Docker container that automatically resolves all installation dependencies (I, R). Comprehensive online material provides full code documentation, architecture description, tutorials, and troubleshooting help (F, A, R). Together with Comprior’s modular architecture with clearly defined interfaces, it supports and encourages researchers to integrate custom extensions into Comprior (A, I, R). In addition, Comprior also returns intermediate data artifacts during the analysis, e.g. the transformed input data set or feature rankings, which can be reused for any other custom workflows (I).

## Implementation

In the following, we discuss Comprior’s technical realization. We first introduce Comprior’s main architecture components. We then describe selected implementation details that introduce extensibility, flexibility, and accessibility into Comprior.

### Architecture design

**Fig. 2:**
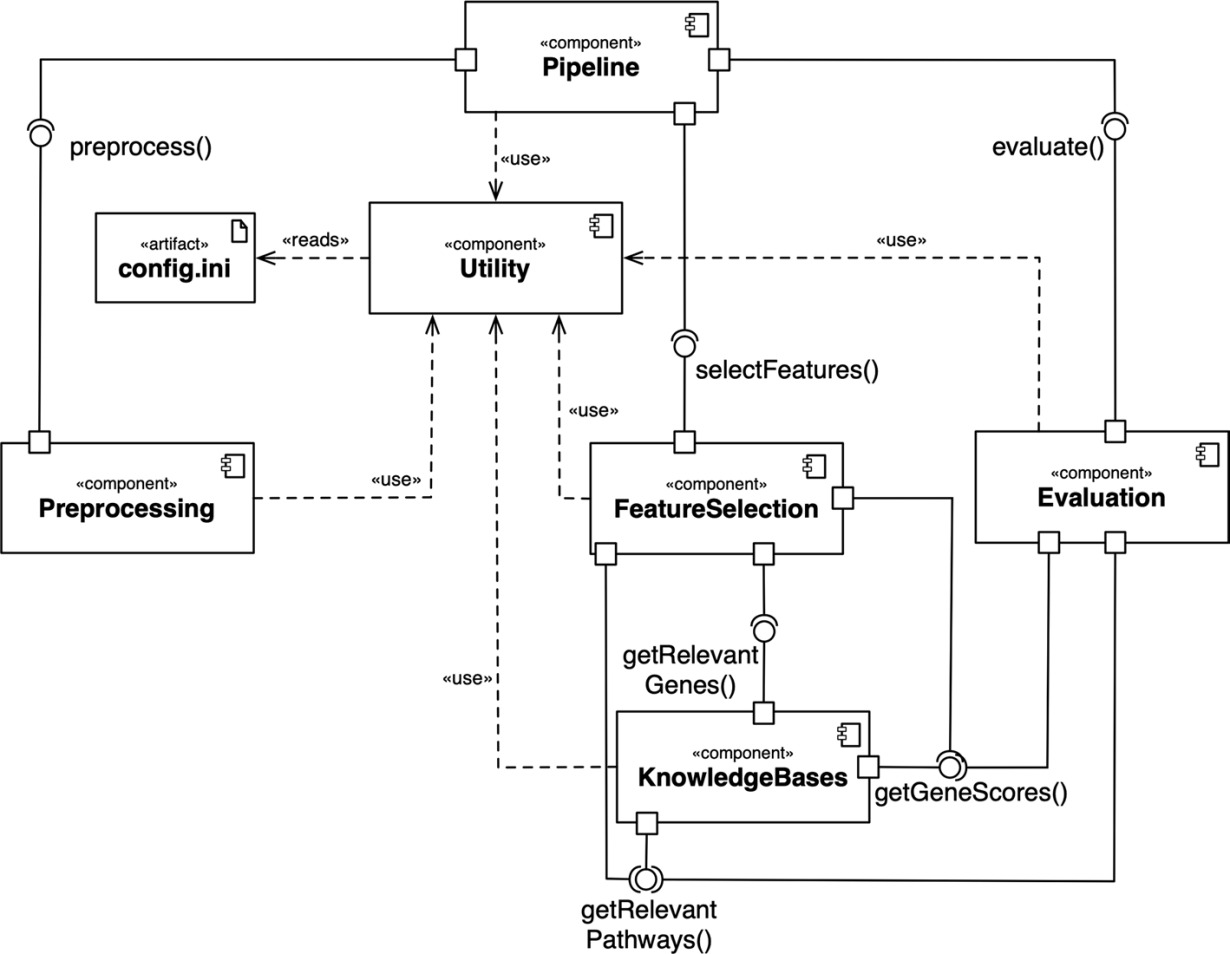
Overview of Comprior’s system components. There is a major Pipeline component for benchmark orchestration, while specific functionality is implemented in dedicated components. Communication between the components is realized via corresponding interface methods

Figure 2 depicts the system architecture of Comprior in a UML 2.0 components diagram. Every component maps to distinct functionality that is needed throughout the benchmarking process.

The **Pipeline** component orchestrates the benchmark execution based on the user-defined configuration: preprocessing the input data, running feature selection approaches, and executing evaluation strategies. The **Utility** component provides general functionality that is needed throughout the whole benchmarking process and is thus accessed by all other modules. It stores configuration parameters, contains functionality for logging, identifier mapping, as well as directory and file management. The **Preprocessing** component is responsible for preprocessing and transforming the input data set, e.g. missing value filtering or identifier mapping. Preprocessing functionality is invoked and organized by the Pipeline component. The **FeatureSelection** component provides the approaches for feature selection. We have implemented feature selectors of different kinds, which

- use traditional approaches from existing packages, e.g. ANOVA,
- provide wrappers that invoke approaches coded in R or Java,
- combine statistical approaches with knowledge bases, and
- select networks, pathways, or submodules as features.

The **KnowledgeBase** component encapsulates implementations of knowledge bases that can be used for information retrieval. Knowledge bases are used by both the FeatureSelection and Evaluation components. The **Evaluation** component encapsulates all functionality for evaluating and assessing input data set quality, knowledge base coverage, and feature selection approaches.

### Extensibility by custom functionality

Comprior was designed to be extensible and facilitate a straightforward implementation of custom approaches. This is achieved by (a) a uniform communication between system components and (b) wrapper functions to include custom functionality from programming languages other than Python.

**Fig. 3:**
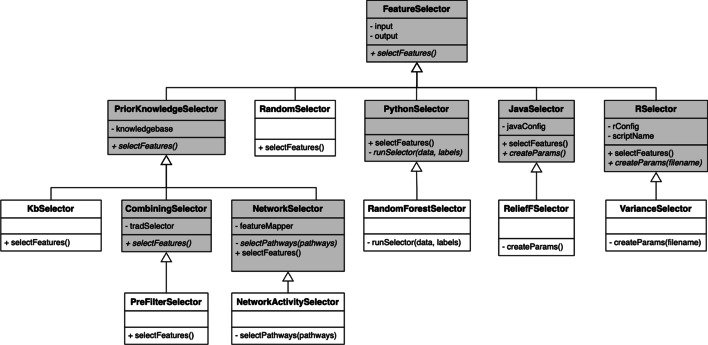
Class structure of the FeatureSelection component (abstract classes in grey). On top of the hierarchy is an abstract FeatureSelector class that defines the method selectFeatures(). This method is invoked during pipeline execution. Actual feature selection strategies are realized in inheriting classes

Comprior realizes a uniform communication via interface methods. If new functionality is integrated, developers must ensure that these interface methods are correspondingly implemented. To further facilitate this, Comprior enforces an inheritance structure with an abstract superclass on top that defines the required interface methods. New functionality must then be implemented in a class that inherits from this superclass and subsequently implements the interface methods. As an example, we outline the class and inheritance structure of the *FeatureSelection* component in Fig. 3. For the sake of clarity, it only shows the most important classes, omitting most of the classes implementing concrete feature selection approaches. Abstract classes that do not implement a specific feature selection approach are shaded in grey. On top of the hierarchy is the main abstract class *FeatureSelector*. All inheriting classes that implement actual feature selection strategies must inherit from it and implement the abstract method *selectFeatures()*, which serves as interface method to the Pipeline component for invoking feature selection. Further inheriting abstract classes provide specialized functionality, e.g. to invoke Java or R code or use Python’s scikit learn (*JavaSelector*, *RSelector*, and *PythonSelector*, respectively). Novel prior knowledge feature selection approaches should inherit from *PriorKnowledgeSelector* or specialized inheriting abstract classes to combine prior knowledge with any existing feature selector (*CombiningSelector*) or to select networks as features (*NetworkSelector*). For the sake of completeness, we refer to the Additional file 1 and Comprior’s documentation site for the detailed class diagrams of all components.

Sometimes, custom functionality must be implemented in a programming language other than Python, e.g. because an efficient implementation is already available or the developer is more familiar with it. While mainly implemented in Python, Comprior allows to invoke non-Python code via wrapper functions. The utility component provides corresponding interface functions for R and Java code, which can easily be extended to other programming languages, e.g. C++^3^.

### Accessibility of prior knowledge

**Fig. 4:**
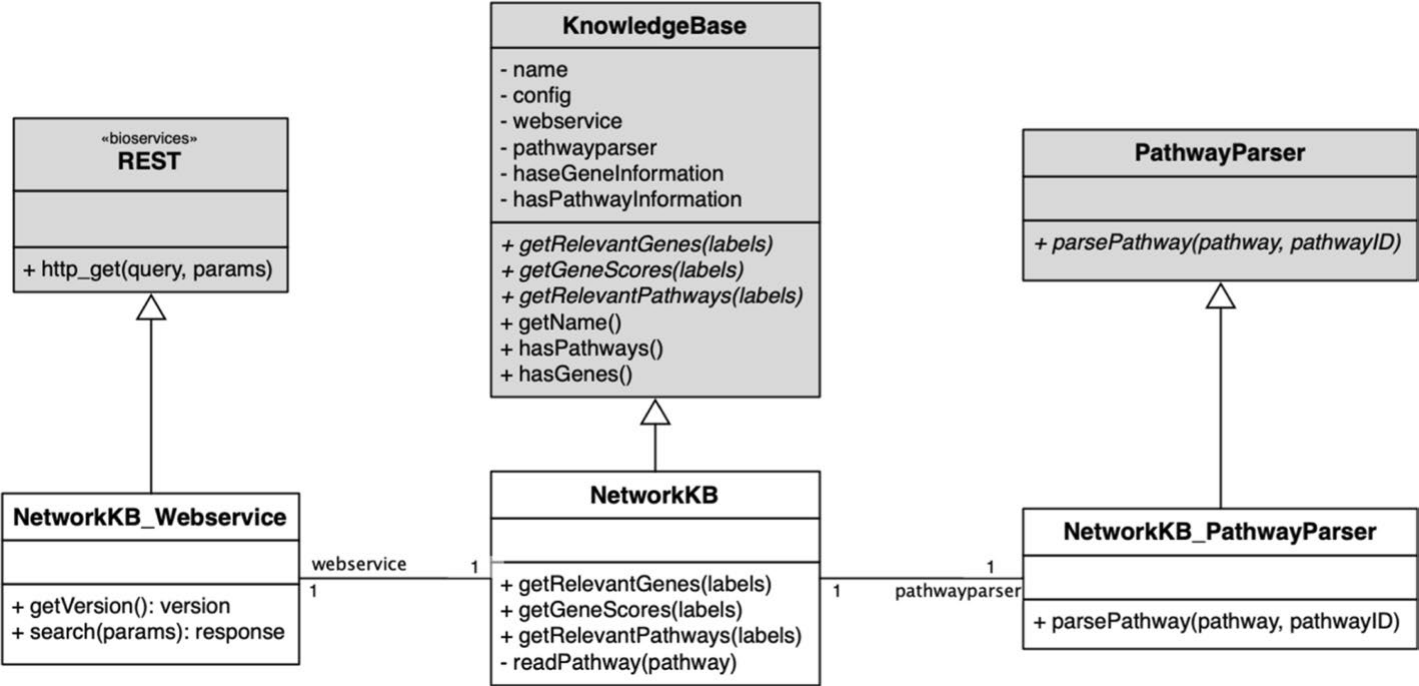
Example implementation of a knowledge base providing network information. Class *NetworkKB* inherits from the abstract *KnowledgeBases* class to implement the required interface functions. Class *NetworkKB_Webservice* retrieves the actual prior knowledge from a web service via their REST APIs. As network information is retrieved, *NetworkKB_PathwayParser* parses the network information and transforms it into a uniform format that is used by Comprior

One of Comprior’s key features is the uniform access to knowledge bases. Researchers who want to implement a new prior knowledge approach and make use of the available knowledge bases do not have to take care of accessing them individually and transforming their result to a uniform format. Figure 4 exemplifies how the concept of a knowledge base is realized in Comprior. Class *NetworkKB* inherits from the abstract *KnowledgeBase* class and interacts with the Pipeline component via the specified interface methods *getRelevantGenes()*, *getGeneScores()*, and *getRelevantPathways()*. A second class *NetworkKB_Webservice* retrieves the actual prior knowledge from the corresponding web service by inheriting from Bioservices’ REST class [25]. Bioservices offer web service query implementations for many biological knowledge bases. If such an implementation is not yet available for a knowledge base, it can be implemented correspondingly. If a knowledge base provides network information, it additionally needs a class inheriting from *PathwayParser* to transform the pathway information to a uniform format that can be used by Comprior. The class uses the Pypath module for parsing pathway information from the knowledge base and transforming it into a network data structure [26]. Pypath provides multiple administrative methods, e.g. for retrieving interaction partners, and even allows to construct a single network from multiple input networks.

If the knowledge base provides network or pathway information only, own strategies for *getRelevantGenes()* and *getGeneScores()* must be implemented as this information does not come from the knowledge base automatically. For pathway information from KEGG and PathwayCommons, Comprior computes a gene score $s_i$ for a gene *i* from the sum of its percentile connectedness ranks $pr_{p, i}$ in a pathway *p*, normalized by the overall number of pathways $P_i$ containing gene *i*:

1 $$\begin{aligned} s_i = \frac{\sum _{p=1}^{|P_i|} pr_{p,i}}{|P_i|} \end{aligned}$$

This way, hub genes with many interactions receive a higher score than genes at the outside rim of a pathway and are even more favored if they are hub genes in multiple pathways.

### Flexible pipeline design

As a benchmark involves multiple processing steps, there are many options for adjustment of each single step. Comprior uses configuration files to enable a flexible pipeline design. There is a main configuration file that specifies all parameters that Comprior needs for functioning properly, including access points to knowledge base web services and output folder structure. On top of that main configuration file, users can specify their own configuration file that contains only those parameters they want to overwrite, e.g. input data or feature selectors.

## Discussion

Comprior supports researchers with various aims: first, those that want to effortlessly implement and benchmark a novel (prior knowledge) feature selection approach without having to deal with cumbersome administrational tasks, e.g. prior knowledge retrieval, cross-validation strategies, or even identifier mapping. Second, Comprior supports those researches that want to analyze gene expression data sets and explore the power of prior knowledge integration, flexibly testing out different knowledge bases and integration levels. With its unified access to prior knowledge, Comprior lowers the bar for integrating it into the analysis of gene expression data and thus facilitates applicability of prior knowledge approaches. By providing both a development and benchmarking tool, prior knowledge approaches can now be easily implemented and thoroughly benchmarked against each other.

### Case study: breast cancer

In a small case study, we demonstrate the usage of Comprior and examine the effectiveness of prior knowledge approaches in terms of classification performance, biological relevance, and robustness. The aim is to identify feature sets for classifying samples of two breast cancer data sets into their PAM50 breast cancer subtypes of luminal A, luminal B, HER2-enriched, basal-like, and normal-like [27, 28]. All figures shown are automatically generated by Comprior.

#### Input data

Comprior expects as input data normalized gene expression levels and corresponding metadata. There are no requirements regarding the file layout, e.g. separators, column orientation, or identifier formats, as Comprior transforms input files automatically as needed. For the case study, we downloaded and preprocessed two breast cancer data sets from The Cancer Genome Atlas (TCGA-BRCA) and Sweden Canceroma Analysis Network-Breast (SCAN-B) initiatives before providing them to Comprior. Descriptions on the conducted preprocessing steps and corresponding R code are provided in the Additional file 1. The final data sets contain 1,090 samples with 20,950 genes (TCGA-BRCA) and 378 samples with 15,011 genes (SCAN-B), respectively.

#### Pipeline setup

Once the input data has been preprocessed, the actual benchmark experiment can be designed by specifying relevant parameters in a configuration file, e.g. where to find the input data, which feature selectors to use, or what performance measurements to plot. It is then provided as input to Comprior when invoking the tool via command line. Comprior reads the parameters from the configuration file, executes the benchmark, and produces summary plots on the results.

The configuration file of this example case study is available on Comprior’s GitHub repository. For prior knowledge retrieval, Comprior uses the class labels of a data set by default. Additionally, users can specify additional search terms as desired via a separate parameter. In this case study, we specified additional search terms related to breast cancer, its PAM50 subtypes, and their corresponding synonyms as looked up in the National Cancer Institute’s metathesaurus browser (https://ncim.nci.nih.gov/ncimbrowser/). For feature selection, we applied ANOVA and corresponding prior knowledge adaptations: prefiltering the input set with relevant genes from OpenTargets or DisGeNET (*Prefiltering_ANOVA_OpenTargets* and *Prefiltering_ANOVA_DisGeNET*) and weighting ANOVA scores by OpenTargets or DisGeNET association scores (*Weighted_ANOVA_OpenTargets* and *Weighted_ANOVA_DisGeNET*). Comprior selects feature sets of increasing size from one to 20 features from the TCGA-BRCA data set. These feature sets are used on both the TCGA-BRCA and the SCAN-B data set for classification. Comprior applies tenfold cross-validation on five different classifiers (Naive Bayes, Linear Regression, Support Vector Machines, Random Forest, and *k*-Nearest neighbor with $k=3$). The final classification performance corresponds to the average classification performance across these classifiers. Comprior uses Enrichr and the *MSigDB_Oncogenic_Signatures* database to assesses the biological relevance of the selected feature sets [29]. For that, Comprior filters terms retrieved by Enrichr with an adjusted *p*-value above 0.05 and then sorts remaining terms in descending order by their combined score.

#### Results

**Fig. 5:**
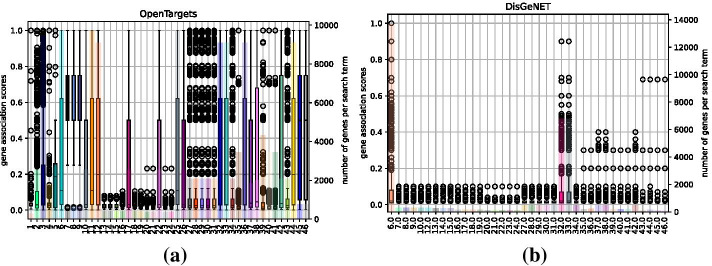
Combined plots showing knowledge base coverage for **a** OpenTargets and **b** DisGeNET. The box plot shows the association scores of relevant genes retrieved (left y axis); the bar plot depicts the overall number of genes retrieved (right y axis)—all grouped by search terms (a mapping from identifier to the actual search term is provided in Additional file 1: Table 1). Both knowledge bases provided results for all search terms, however OpenTargets provided more genes with higher association scores than DisGeNET

Figure 5 shows the coverage of search terms in both OpenTargets and DisGeNET. A mapping of identifiers used in the diagrams and the actual search term is provided in Additional file 1: Table 1. While both knowledge bases provided prior knowledge for all 46 search terms, OpenTargets generally returned both a higher number of associated genes and higher association scores. Association scores returned by DisGeNET are generally low, while being moderate for OpenTargets. From the point of knowledge base coverage, we thus expect adaptations using OpenTargets to achieve better performance than those using DisGeNET.

**Fig. 6:**
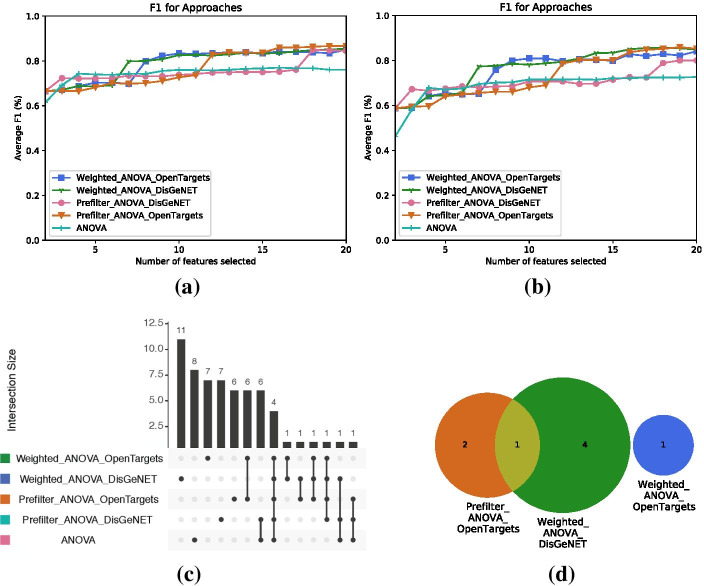
Performance results showing **a** $F_1$ classification performance for one to 20 features on the TCGA-BRCA data set, **b** $F_1$ classification performance for the same feature sets on the SCAN-B data set, **c** overlaps of the top 20 features selected by the different approaches, and **d** overlaps of the top 20 enriched terms from the feature sets ($n=20$) as Upset diagrams

Figure 6a depicts $F_1$ classification performance on the TCGA data set for feature set sizes between one and 20 features. These features are used to classify the SCAN-B data set, for which $F_1$ classification performance is shown in Fig. 6b. All prior knowledge adaptations of ANOVA perform better than the original approach. While $F_1$ performances for all adapted approaches reach a plateau around 0.83 at 17 features on the original data set, weighted approaches reach this plateau earlier around seven selected features, while the prefiltering approaches require 12 and 18 features. The integration of prior knowledge further improves the robustness of the feature sets, as the adapted approaches generally show better $F_1$ scores on the cross-validation data set.

Figure 6c depicts overlaps of feature sets ($n = 20$) selected by the different approaches. All approaches agreed on 25% of the features, but also selected between 30 and 55% distinct features. Approaches using OpenTargets share 60% of their features, while such a high overlap does not occur for approaches using DisGeNET. This may be related to the lower coverage of the applied search terms in DisGeNET. Figure 6d depicts overlaps of the enriched oncogenic signatures from MSigDB for the feature sets ($n = 20$). Feature sets from both ANOVA and Prefiltering it with DisGeNET were not enriched with any oncogenic signature. Weighting ANOVA scores by DisGeNET association scores resulted in the highest number of oncogenic signatures. Still, only two of the adapted approaches share a single oncogenic signature at all. At this point, further investigation on the concrete oncogenic signatures and their relations would be necessary. However, we leave this task for future work as this is not in the scope for demonstrating Comprior’s usability.

### Functional comparison to other tools

Multiple software tools have been developed to enable reproducible benchmarking in the bioinformatics domain. Table 1 provides a functional comparison between Comprior and the state of the art.

**Table 1:** Functional comparison of existing benchmarking tools for analyzing gene expression data

Approach	Domain	Benchmarking pipeline parts			Standard approaches	Exten-sibility	Prior knowledge	Evaluation functionality				
		Flexible design	Automated execution	Result analysis				Standard metrics	Runtime	Biological relevance	Cross-validation	Visualization
iCOBRA [30]	Ranking comparison, binary assignments, e.g. DEA			$\bullet$				$\bullet$				$\bullet$
ClassifyR [3]	Gene expression feature selection, classification	($\bullet$)	$\bullet$	$\bullet$	$\bullet$	$\bullet$		$\bullet$			$\bullet$	$\bullet$
DaMiRseq [1]	Gene expression feature selection, classification		$\bullet$	$\bullet$	$\bullet$			$\bullet$			$\bullet$	$\bullet$
OmicsMarkeR [2]	Omics feature selection, classification	($\bullet$)	$\bullet$	$\bullet$	$\bullet$			$\bullet$			$\bullet$	
Comprior	Gene expression feature selection, classification	($\bullet$)	$\bullet$	$\bullet$	$\bullet$	$\bullet$	$\bullet$	$\bullet$	$\bullet$	$\bullet$	$\bullet$	$\bullet$
NormalyzerDE [31]	Gene expression normalization, DEA	($\bullet$)	$\bullet$	$\bullet$	$\bullet$	$\bullet$		$\bullet$				$\bullet$
Summarized-Benchmarks [32]	General purpose	$\bullet$	$\bullet$	$\bullet$		$\bullet$		$\bullet$				$\bullet$
CellBench [33]	General purpose	$\bullet$	$\bullet$	$\bullet$		$\bullet$			$\bullet$			
pipeComp [34]	General purpose	$\bullet$	$\bullet$	$\bullet$		$\bullet$		$\bullet$	$\bullet$			$\bullet$

The tools cover different parts of the benchmarking process and generally focus on result assessment based on standard performance metrics, e.g. accuracy. While the general purpose tools provide highest flexibility regarding pipeline design and extensibility, they do not provide built-in standard approaches for benchmarking. On the contrary, tools providing built-in approaches are typically not extendable. Comprior provides both built-in approaches and extensibility and is furthermore the only tool that focusses on prior knowledge retrieval and result set assessment regarding biological relevance

When comparing general purpose tools and those specialized on gene expression analyses, both extensibility and flexibility in pipeline design typically come at the cost of missing built-in approaches and many administrational tasks, e.g. cross-validation. While general purpose tools allow users to design their pipeline with any desired tool, they have to come up with the functionality needed by the pipeline on their own: approaches for comparisons, cross-validation strategies, or even simple but cumbersome administrational tasks like identifier mapping are usually not supplied. On the contrary, specialized tools provide this functionality to users, allowing them to choose from a range of built-in standard approaches when designing their pipeline. However, most of these tools are not meant to be extended by custom functionality, rendering them impractical for testing custom approaches. Nearly all of the compared tools provide some standard evaluation metrics, e.g. ROC, and corresponding visualizations. Most of the tools assess benchmark results only by these metrics; few tools provide runtime measurements; none of them incorporates biological knowledge from public resources, neither for assessing the biological relevance of the results, e.g. via enrichment analysis, nor for integrating it during the actual analysis.

Comprior fills these gaps as it provides a broad range of built-in standard approaches - covering both statistical feature selection and prior knowledge approaches - and maintaining extensibility at the same time. What is more, Comprior enables a more comprehensive result assessment that covers standard performance metrics, e.g. accuracy, runtime performance, and biological relevance.

### Future work

For the future, we will further extend Comprior’s functionality focussing on the distinct processing steps. In particular, we plan to integrate normalization strategies for preprocessing and a prediction component for subsequent analysis, which also involves the generation of further visualizations. From a technical point of view, refactoring of the classification component will provide benefits as it reduces code heterogeneity. When Comprior was initially built, no Python wrapper existed for WEKA, on which our classification component relies [35]. Such wrappers are now available and we plan to implement the classification component completely in Python.

## Conclusion

Comprior is a benchmarking tool for feature selection approaches and specifically addresses the needs of prior knowledge approaches. It supports the complete benchmarking process from pipeline design to execution and result set visualization. Comprior provides cross-validation strategies for examining the robustness of feature selection approaches. What is more, Comprior supports annotation and enrichment of feature sets to assess and compare their biological relevance. Users are flexible in pipeline design as they can choose from a broad range of both statistical and prior knowledge feature selection approaches, classifiers, knowledge bases, and performance measures. At the same time, Comprior is designed to be efficiently and effortlessly extensible by custom functionality, which constitutes a meaningful enhancement of the current state of the art.

## Availability and requirements

Project name: Comprior

Project home page: Code documentation, technical specifications, tutorials, and how-tos are available at https://comprior.readthedocs.io/en/latest/

Code Availability: Complete code for download available at GitHub: https://github.com/CPerscheid/Comprior

Operating system(s): Platform independent

Programming language: Python, R, Java

Other requirements: For running Comprior out of the box: Docker. For installing and running Comprior from source: R 3.5 or higher, Python 3.5 or higher, Java 1.8 or higher, Maven.

License: MIT

Any restrictions to use by non-academics: No restrictions.

## Supplementary Information

**Additional file 1.** Supplementary material describing data preprocessing and providing a mapping table for applied search terms.

## Data Availability

The datasets generated and/or analysed during the current study are available in the Comprior repository, https://github.com/CPerscheid/Comprior.

## Footnotes

1 for the complete list, please visit the documentation https://Comprior.readthedocs.io/en/latest/configparams.html

2 for a more detailed description, please visit the documentation https://comprior.readthedocs.io/en/latest/priorknowledge.html

3 for a corresponding tutorial, please visit https://comprior.readthedocs.io/en/latest/howtos.html#add-custom-code-from-r-java-another-programming-languages
