# Supplementary material for: Comprior: facilitating the implementation and automated benchmarking of prior knowledge-based feature selection approaches on gene expression data sets
Source: BMC Bioinformatics. 2021 Aug 12;22:401. doi: 10.1186/s12859-021-04308-z (PMC8361636; doi:10.1186/s12859-021-04308-z)
Supplement: Supplementary file 1 — Additional file 1. Supplementary material describing data preprocessing and providing a mapping table for applied search terms. [file 12859_2021_4308_MOESM1_ESM.pdf]

Supplementary Material - Case Study

Cindy Perscheid

1 Dataset Preprocessing

The *TCGA* data set was originally acquired in the scope of the TCGA-BRCA program. We downloaded raw expression counts of primary tumor tissue and corresponding metadata information from the NCI Genomic Data Commons public database. From the downloaded data, we removed all samples without PAM50 subtype assignment. We further filtered genes that had less than 60 read counts in more than 30 percent of a class' samples and applied TMM normalization with subsequent log2-transformation. The final data set contains 1,090 samples and 20,950 genes. Listing 1 provides the corresponding R code.

```
library(TCGAbiolinks)
library(edgeR)
library(SummarizedExperiment)


home.folder <- "/path/where/to/store/the/data/"
########### BRCA ##########
brca.project <- c("TCGA-BRCA")
brca.subtypeColumn = "subtype_BRCA_Subtype_PAM50"
brca.fileprefix = paste0(home.folder, "BRCA_TP_")
brca.metadata.attributes = c("sample","patient","barcode","
    shortLetterCode","definition","tumor_stage","classification_of_
    tumor","tumor_grade","primary_diagnosis","age_at_diagnosis","
    gender","disease_type","primary_site","project_id","subtype_
    Tumor.Type","subtype_Included_in_previous_marker_papers","
    subtype_Tumor_Grade","subtype_BRCA_Pathology","subtype_BRCA_
    Subtype_PAM50","subtype_MSI_status","subtype_CNV.Clusters","
    subtype_Mutation.Clusters","subtype_DNA.Methylation.Clusters","
    subtype_mRNA.Clusters","subtype_miRNA.Clusters","subtype_lncRNA
    .Clusters","subtype_Protein.Clusters")

#get only primary tumor samples
TCGAquery <- GDCquery(project= brca.project, data.category = "
    Transcriptome Profiling", data.type = "Gene Expression
    Quantification", workflow = "HTSeq - Counts", barcode = c(""),
    sample.type = c("Primary solid Tumor"))

#download the actual data
GDCdownload(TCGAquery)
#prepares the data for analysis and putting it into an
    SummarizedExperiment object
expr1 <- GDCprepare(TCGAquery)

```

```r

#get the metadata
metadata <- colData(expr1)
expr.subtype <- metadata[,c("sample", brca.subtypeColumn)]

#remove NAs
has.subtype <- expr.subtype[!is.na(expr.subtype[[brca.subtypeColumn]]),]

#subset only to the patients with available subtype information
expr.subset <- subset(expr1, select = colData(expr1)$sample %in% has.subtype$sample)

#remove unclassified samples
conditioned <- row.names(metadata[!is.na(metadata[[brca.subtypeColumn]]),])
intersection <- intersect(colnames(expr.subset), conditioned)
expr <- expr.subset[,intersection]

#create DGEList object
genes <-rowRanges(expr)$external_gene_name
expr.subtype <- colData(expr)[[brca.subtypeColumn]]
expr.counts <- assay(expr)
expr.dge <- DGEList(expr.counts, group=expr.subtype, genes=genes)

design <- model.matrix(~ factor(expr.subtype))

keep <- filterByExpr(expr.dge, design, min.count = 60)
expr <- expr.dge[keep,]

#normalize counts with TMM
expr.normalized <- calcNormFactors(expr, method = "TMM")
expr.normalized.matrix <- log2(cpm(expr.normalized) + 1)

#write metadata
metadata <- colData(expr.subset[row.names(expr.normalized.matrix)])
metadata.filtered <- metadata[,brca.metadata.attributes]
write.table(metadata.filtered, file=paste0(brca.fileprefix, "metadata.csv"), sep = ";")

#write counts data
write.table(expr.normalized.matrix, file=paste0(brca.fileprefix, "expressions_normalized.csv"), sep = ";")
```

Listing 1: R script for downloading and preprocessing TCGA BRCA dataset

The *SCAN-B* data set was acquired within the smaller cohort of the SCAN-B study. We downloaded the data from Gene Expression Omnibus (GEO, GSE81538), for which FPKM log2-transformed expression levels were available. We conducted a principal component analysis (PCA) and removed outlier samples with a principal component value below -800 as shown in Figure 1. As FPKM values are not suitable for comparisons across samples, we transformed these to zFPKM values. For that, we first reverse-transformed expression levels to retrieve the original FPKM values and subsequently applied zFPKM normalization. We then filtered genes that had a zFPKM score score above -3.0 in more than 30 percent of the samples. As we use the SCAN-B data set for cross

validation, we used Comprior's preprocessing functionality to label the data set with the cancer subtypes from the metadata. The final data set contains 378 samples and 15,011 genes. Listing 2 provides the corresponding R code.

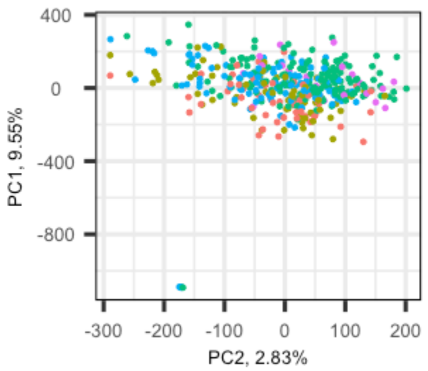

Figure 1: Principal component analysis of the SCAN-B data set. We removed outliers with a pc1 value below -800 from the data set.

```
library(genefilter)
library(GEOquery)
library(zFPKM)
library(PCAtools)


home.folder <- "/path/where/to/store/the/data/"
file.prefix <- "BRCA/SCANB"
accession.number <- "GSE81538"

#get expression data
data <- getGEO(accession.number, GSEMatrix=TRUE)
filePaths = getGEOSuppFiles(accession.number)
expr.filename <- row.names(filePaths)[2]
expr <- read.csv(expr.filename, row.names = 1)

#add sample IDs as colnames (we assume that the order remains the same)
colnames(expr) <- colnames(data[[1]]@assayData[["exprs"]])

#metadata
metadata <- data[[1]]@phenoData@data

#remove NA samples
expr <- expr[, colSums(is.na(expr)) != nrow(expr)]

#remove unclassified samples
conditioned <- row.names(metadata[!is.na(metadata[["pam50 subtype:ch1"]]),])
intersection <- intersect(colnames(expr), conditioned)
expr <- expr[,intersection]
metadata <- metadata[intersection,]
```

```r

#reverse-transform from log2-FPKM and subtract 0.1 to have original FPKM values
#excerpt from study: "...adding to each expression measurement 0.1 FPKM, performing a log2 transformation."
exp.fpkm <- 2^expr
exp.fpkm.original <- exp.fpkm - 0.1
exp.zfpkm <- zFPKM(exp.fpkm.original)

#make a PCA and see if we have outliers
p <- pca(exp.zfpkm, metadata = metadata)

#optional: plot PCA
#pairsplot(p, colby="pam50 subtype:ch1")

exp.zfpkm.nooutliers <- exp.zfpkm[,(p$rotated$PC1 > -800)]

#match sample ids for expression and metadata again
intersection <- intersect(colnames(exp.zfpkm.nooutliers), conditioned)
exp.zfpkm.nooutliers <- exp.zfpkm.nooutliers[,intersection]
metadata <- metadata[intersection,]

#filter out lowly expressed genes via genefilter
thres <- (ncol(exp.zfpkm.nooutliers) * 30) / 100
#filter all expression values that have zfpkm score above -3.0 in more than 30% of the samples
expr.zfpkm.nooutliers.filtered <- exp.zfpkm.nooutliers[(rowMedians(as.matrix(exp.zfpkm.nooutliers)) > -3.0), ]

#put genes into columns
expr.zfpkm.nooutliers.filtered <- t(expr.zfpkm.nooutliers.filtered)

expr.prep.filename <- paste0(home.folder, file.prefix, "_normalized_expressions.csv")

#save with genes in columns
metadata <- metadata[,c("title","geo_accession", "pam50 subtype:ch1")]
colnames(metadata) <- c("title", "geo_accession", "pam50_subtype:ch1")

write.table(expr.zfpkm.nooutliers.filtered, file= expr.prep.filename, sep = ";")

#write metadata with samples in columns
write.table(t(metadata), file=paste0(home.folder, file.prefix, "_metadata.csv"), sep = ";")
```

Listing 2: R script for downloading and preprocessing SCANB dataset

2 Knowledge Base Coverage

ID	Search Term	ID	Search Term
1	LumB	25	Normal Breast-Like Subtype of Breast Cancer
2	Basal	26	Normal Breast-Like Subtype of Breast Carcinoma
3	LumA	27	Invasive Breast Cancer
4	Her2	28	Invasive Breast Carcinoma
5	Normal	29	Infiltrating Breast Cancer
6	Breast Cancer	30	Infiltrating Breast Carcinoma
7	ERBB2 Overexpressing Subtype of Breast Carcinoma	31	Invasive Mammary Carcinoma
8	HER2 Overexpressing Breast Carcinoma	32	Mammary Carcinoma
9	HER2 Positive Breast Cancer	33	Breast Carcinoma
10	HER2 Positive Breast Carcinoma	34	Infiltrating Carcinoma of Breast
11	HER2+ Breast Cancer	35	Breast Ductal Carcinoma
12	Human Epidermal Growth Factor 2 Positive Carcinoma Of Breast	36	Mammary Ductal Carcinoma
13	Basal-Like Breast Cancer	37	Duct Adenocarcinoma
14	Basal-Like Breast Carcinoma	38	Duct Carcinoma
15	Basal-Like Subtype of Breast Carcinoma	39	Ductal Adenocarcinoma
16	Luminal A Breast Cancer	40	Ductal Carcinoma of Breast
17	Luminal A Breast Carcinoma	41	Ductal Breast Carcinoma
18	Luminal A Estrogen Receptor Positive Subtype of Breast Carcinoma	42	Ductal Carcinoma
19	Luminal A	43	Lobular Carcinoma
20	Luminal B	44	Infiltrating Lobular Carcinoma of Breast
21	Luminal B Breast Cancer	45	Lobular Adenocarcinoma
22	Luminal B Breast Carcinoma	46	Lobular Breast Carcinoma
23	Luminal B Estrogen Receptor Positive Subtype of Breast Carcinoma		
24	Luminal B Subtype of Breast Carcinoma		

Table 1: ID mapping of applied search terms
